# Effects of Aging on Expression of *Mic60* and *OPA1* and Mitochondrial Morphology in Myocardium of Tibetan Sheep

**DOI:** 10.3390/ani10112160

**Published:** 2020-11-20

**Authors:** Guan Wang, Yuzhu Luo, Jiang Hu, Jiqing Wang, Xiu Liu, Shaobin Li

**Affiliations:** 1 Gansu Key Laboratory of Herbivorous Animal Biotechnology, Faculty of Animal Science and Technology, Gansu Agricultural University, Lanzhou 730070, China; 13680740a@sina.com (G.W.); huj@gsau.edu.cn (J.H.); wangjq@gsau.edu.cn (J.W.); liuxiu@gsau.edu.cn (X.L.); 2 School of Biotechnology and Food, Shangqiu Normal University, Shangqiu 476000, China

**Keywords:** aging, *Mic60*, *OPA1*, myocardial mitochondria, Tibetan sheep

## Abstract

**Simple Summary:**

Mitochondria play a crucial role in the adaptation to high altitude hypoxia environment in Tibetan sheep, and the changes of its morphology and structure directly affect its function. *OPA1* and *Mic60* are important mitochondria-shaping proteins that work together to regulate the morphology of mitochondrial inner membrane and cristae. It has been shown that aging affects the expression of *OPA1* and *Mic60* in mice, but it has not been investigated in sheep and hence it is not known whether it might affect the ultrastructure of mitochondria. In this context, reverse transcription-quantitative PCR (RT-qPCR), enzyme-linked immunosorbent assay (ELISA) and immunohistochemistry method were used to measure the expression of *Mic60* and *OPA1* genes and proteins in myocardium of adult and aged Tibetan sheep, and the ultrastructure of mitochondria were compared by transmission electron microscope. The results suggest that aging can reduces the expression of *Mic60* and *OPA1* genes and *OPA1* protein, which can affect the mitochondrial function.

**Abstract:**

In order to investigate the effects of aging on the expression of *Mic60* and *OPA1* and mitochondrial morphology in plateau animals, the expression of *Mic60* and *OPA1* genes and proteins, and the morphology of mitochondria in the myocardium of adult and aged Tibetan sheep were investigated. The expression of *Mic60* and *OPA1* genes and *OPA1* protein were higher (*p* < 0.05) in the myocardium of adult Tibetan sheep than in those of the aged ones. The number of mitochondrial cristae in the myocardium of adult was higher than that in aged (*p* < 0.05). The density of mitochondria in the myocardium of adult was higher than that in aged (*p* < 0.01). Compared with the adult Tibetan sheep, the mitochondrial crista of aged were relatively sparse, the crista membrane was wide, and the mitochondria were not closely linked, showing fragmentation. These results suggest that the myocardial mitochondria of the adult have better energy supply ability, indicating that aging can lead to the weakening of oxygen supply in the myocardial mitochondria of Tibetan sheep.

## 1. Introduction

Tibetan sheep live in the Qinghai-Tibet plateau and its adjacent areas at an altitude of 3000 to 5000 m above sea level. Generation reproduction under the altitude environment makes Tibetan sheep well adapted to the low oxygen concentration. It also makes it a good model experimental animal for cardiovascular research. The heart is one of the most important organs in animals, which is the power center for the supply of oxygen in whole body tissues. Under increased metabolism and high oxygen consumption, the heart can provide pressure to increase the blood flow into all parts of the body. As an aerobic tissue, the energy of myocardial metabolism is almost entirely dependent on the oxidative phosphorylation of mitochondria. Therefore, the quantity, function and structure of mitochondria in cardiomyocytes determine the energy supply of the heart.

Mitochondria are the main sites for aerobic respiration of eukaryotic cells and play an important role in the maintenance of calcium homeostasis and the regulation of apoptosis [1,2]. The morphology of mitochondria is regulated by fusion and fission [3,4]. The complete morphological structure is the basis for the function of mitochondria and the integrity of the crista membrane is particularly important [5]. Mitochondrial crista membrane is the main site of oxidative phosphorylation. More than 90% of mitochondrial oxidative phosphorylation complex III, ATP synthase and many proteins and enzymes associated with the oxidative phosphorylation are located in crista [6,7,8].

Mitochondria-shaping proteins *Mic60* (Mitofilin) and *OPA1* (Dominant Optic Atrophy), which are located in the mitochondrial membrane space, play an important role in the maintenance of mitochondrial morphology and crista membrane structure [9,10]. The nuclear gene *OPA1* is widely expressed in many organs, but the expression level is different. The main functions of *OPA1* is to promote mitochondrial fusion [11,12]. Inhibition of *OPA1* gene expression or inactivation of *OPA1* protein will lead to the disruption of mitochondrial network fusion [13]. The loss of *OPA1* subtype will cause damage to the crista membrane structure and seriously affects cell proliferation [14,15]. Mic60, as the core component of MINOS (Mitochondrial Contact Site) complex [16], plays an important role in maintaining the morphology and structure of mitochondrial crista [17]. Interfering with the expression of *Mic60* not only leads to mitochondrial malformation, but also inhibits the mitochondrial oxidative phosphorylation activity and the normal function of mitochondria [18]. Recent studies showed that *Mic60* was crucial for the formation of mitochondrial crista junctions [19,20].

The change of heart function is closely related to age [21]. The age-related changes of cardiac myocardium focus on three main aspects: myocardial cells, mitochondria related proteins and enzymes, and collagen fibers. In this study, the effects of aging on the expression of *Mic60* and *OPA1* genes and proteins and the mitochondrial morphology in myocardial of Tibetan sheep were studied in combination with the observation of molecular regulation mechanism, protein expression, and mitochondrial ultrastructure.

## 2. Materials and Methods

All animal work in this study was conducted according to the guidelines of the care and use of experimental animals established by the Ministry of Science and Technology of the People’s Republic of China (Approval number 2006-398), and was approved by the Animal Care Committee of Gansu Agricultural University.

### 2.1. Materials

Six ewes of Tibetan sheep in Gannan Luqu (the average altitude is 3500 m) were selected randomly, three adult (2 years old) and three aged (6 years old) each. All ewes were slaughtered according to the conventional methods, and the same site of myocardial tissue was immediately collected. Of these, 200 mg was immediately used for the isolation of the mitochondria. The sample for light microscopy was cut into pieces of 1 × 1 × 1 cm, washed with PBS and fixed in a 4% paraformaldehyde fixative solution for later use. Transmission electron microscope samples were cut into strips of 1 × 1 × 2 mm, washed with PBS and fixed in 3% glutaraldehyde phosphate buffer (pH 7.2–7.4, 4 °C). The rest of the samples was rapidly frozen by liquid nitrogen and then stored in the refrigerator at −80 °C for the determination of *Mic60* and *OPA1* genes and proteins expression.

### 2.2. Isolation of the Myocardial Mitochondria

Mitochondria in myocardial tissue were isolated according to the Biovision mitochondrial isolation kit (Mammalian Mitochondria Isolation Kit for Tissue and Cultured Cells, K288). The whole process of isolation was carried out at low temperature to ensure the complete structure and function of mitochondria. The isolated mitochondria were fixed with 3% glutaraldehyde phosphate buffer (pH 7.2–7.4, 4 °C).

### 2.3. Extraction of Tissue RNA

The total RNA in myocardium was extracted according to the RNA extraction kit (RNA isolator Total RNA Extraction Reagent, Vazyme, Nanjing, China). The quality of RNA integrity was tested by 1.5% agarose gel electrophoresis. Next, the OD260/OD280 value and its concentration were measured by ultramicro spectrophotometer, and the qualified RNA was preserved in the freezer at −80 °C. Reverse transcription of RNA was completed by using reverse transcription kit (HiScript ⅡQ RT SuperMix for qPCR, +gDNA wiper, Vazyme, Nanjing, China). The extracted cDNA was diluted into 1:9 solution and stored at −20 °C.

### 2.4. Design and Synthesis of Primers

The complete sequence of sheep *Mic60* and *OPA1* genes published by GenBank were used to design PCR primers by Primer Premier 5.0 software (PREMIER Biosoft, San Francisco, CA, USA). The β-actin gene was used as the reference gene, and the designed primers were tested for its specificity by Blast. Primers were synthesized by BGI Liuhe Company (Beijing, China). The detail information of primers is shown in Table 1.

### 2.5. RT-qPCR Reaction

The cDNA working fluid was carried out by SYBR Green I chimeric fluorescence method using ChamQ Universal SYBR qPCR Master Mix (Q711-02/03, Vazyme, Nanjing, China) for RT-qPCR reaction. The 20 μL reaction containing 2 μL of cDNA working fluid, 0.4 μL of upstream and downstream primers (10 μM), respectively, 2× ChamQ Universal SYBR qPCR Master Mix (10 μL) and RNase-free dd H_2_O (7.2 μL). The optimal conditions of qPCR amplification procedure were as follows: Pre-denaturation at 94 °C for 4 min; Cyclic reaction, 95 °C 10 s, 60 °C 30 s, 40 cycles; Dissolving curve, 95 °C 15 s, 60 °C 60 s, 95 °C 15 s. The specificity of the PCR product and the presence of primer dimers were determined according to the resulting dissolution curve. Data were collected and gene expression was calculated [22] and analyzed for variance using SPSS software (SPSS, Chicago, IL, USA).

### 2.6. Immunohistochemistry and ELISA

The samples of cut sections were fixed with 4% paraformaldehyde fixative (4 °C, 24 h) and then cut into 5 μm thick sections by routine paraffin embedding. Cut sections were deparaffinized to water for antigen retrieval (Citrate Buffer-Microwave Heat Recovery Method). After rinsed in PBS, the cut sections were blocked (37 °C, 10 min) by adding 3% H_2_O_2_ (SP KIT), rinsed in PBS, placed in wet box and added blocking solution (SP KIT A) on it (at room temperature, 10 min), and then incubated overnight at room temperature with primary antibody (Rabbit Anti-Mitofilin antibody and Rabbit Anti-*OPA1* antibody, 1:200, Bioss, Beijing, bs-11764R and bs-1824R). The sections were rinsed by PBS, and then added the secondary antibody (SP KIT B) on it (37 °C, 15 min). The sections were rinsed by PBS and the third antibody (SP KIT C) was added (37 °C, 15 min). At last, the sections were stained by DAB, slightly stained by hematoxylin and fixed by neutral balata. An Olympus BX61 positive universal microscope (Olympus, Tokyo, Japan) was used to observe the sections. The *Mic60* and *OPA1* proteins in the myocardium were detected by ELISA. To measure this, 100 mg of fresh tissue was triturated using a homogenizer (Biospec, Bartlesville, OK, USA) and mixed with 500 µL PBS. The solution was then centrifuged for 10 min at 3000× *g* at 4 °C and the supernatant collected. The protein was measured and a solution containing 100 µg proteins was analyzed according to the manufacturer’s instructions (Mic60 and *OPA1* ELISA Kit, Bio-Swamp, Wuhan Beinglay Biotech Co., LTD, Wuhan, China). The PBS solution used for grinding tissue was analyzed as blank control and the absorbance was measured at 450 nm. The concentrations of *Mic60* and *OPA1* were calculated from corresponding with the *Mic60* and *OPA1* standard curves.

### 2.7. Transmission Electron Microscopy Preparation

The tissue and isolated mitochondria for cut sections were fixed with 3% glutaraldehyde, rinsed twice with 0.2 mmol/L PB (10 min/time), fixed with 1% osmium tetroxide (4 °C, 2 h), and rinsed three times with PB (10 min/time). Then, the samples were dehydrated by gradient alcohol (50, 60, 70, 80, and 90% ethanol for 10 min and 100% acetone dehydration twice for 10 min each). The samples were soaked in the mixture of epoxy resin (SPION-PON812) with acetone (1:1) for 5 h (room temperature), embedded with epoxy resin and dried (45 °C, 12 h; 65 °C, 48 h). Semi-thin sections were cut before making ultrathin sections (70 nm). The ultrathin sections were stained by uranium and lead (Uranium acetate stained for 30 min and lead citrate stained for 10 min). The ultrathin sections were observed using a JEM-1230 transmission electron microscope (Jeol, Nihon Denshi, Japan).

### 2.8. Statistical Analyses

To measure the number of mitochondrial crista and density of mitochondria, 50 microscopic fields (10 cut sections were made for each animal and 5 microscopic fields were randomly selected from each cut section, 20,000×) were analyzed from each animal included in the study. All data in this study were analyzed by Independent Samples t-test using SPSS 19.0 (SPSS, Chicago, IL, USA) and were expressed as the mean ± SD. All *p*-values were considered statistically significant when *p* < 0.05.

## 3. Results

### 3.1. Analysis of the Result of RT-qPCR

**Figure 1:**
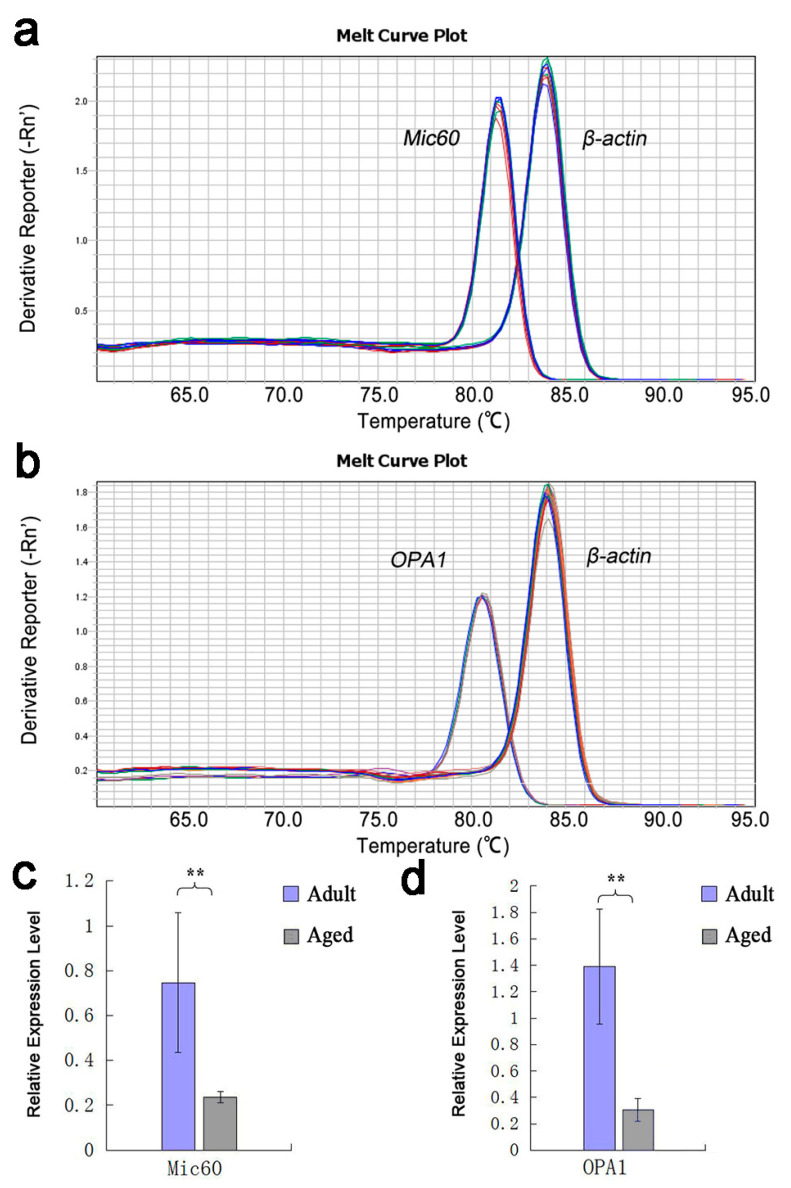
Melting curves of *Mic60*, *OPA1* and β-actin genes and their relative expression in adult and aged Tibetan sheep cardiomyocytes. Note: (**a**,**b**): Melting curve of *Mic60*, *OPA1* and β-actin genes. (**c**,**d**): Relative expression of *Mic60* and *OPA1* genes. All data showed as means ± SD (*n* = 3). ** *p* < 0.01.

The expression of *Mic60* and *OPA1* genes in myocardium of adult and aged Tibetan sheep was quantitatively analyzed by RT-qPCR. The results showed that both the *Mic60* and *OPA1* genes were expressed in the myocardium of adult and aged Tibetan sheep, but the expression levels were different. The Tm values of the two gene amplification products were uniform, and the single peak melt curve indicated that both primer sets had good specificity in the RT-qPCR reaction, and no non-specific amplification and primer dimers were observed (Figure 1a,b). The relative expression levels of *Mic60* and *OPA1* genes in the myocardium of adult Tibetan sheep were 0.7471 ± 0.3105 and 1.3922 ± 0.43547, respectively, which were significantly higher than those in aged Tibetan sheep (0.2366 ± 0.02534 and 0.307 ± 0.08628, respectively) (*p* < 0.01) (Figure 1c,d).

### 3.2. Expression and Analysis of Mic60 and OPA1 Proteins

**Figure 2:**
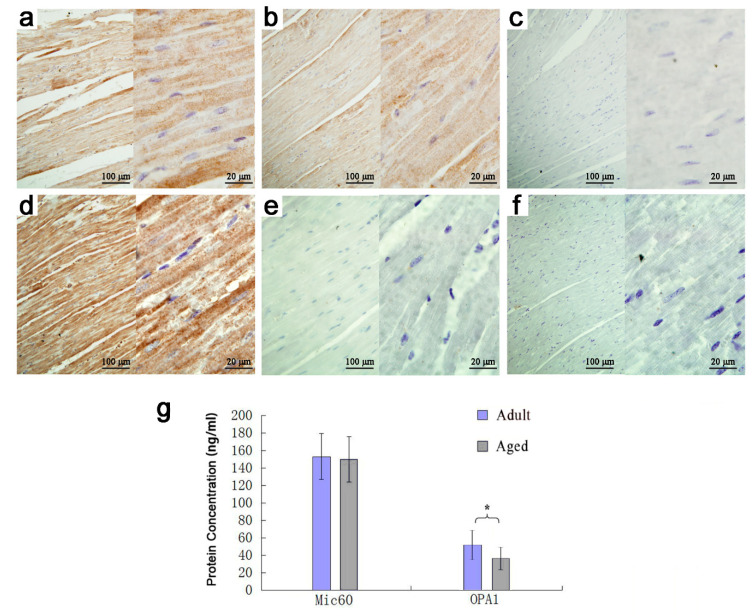
Expression of *Mic60* and *OPA1* proteins in cardiomyocytes. Note: (**a**–**c**): Expression of *Mic60* protein in adult and aged Tibetan sheep myocardium and blank control. (**d**–**f)**: Expression of *OPA1* protein in adult and aged Tibetan sheep myocardium and blank control. (**g**): Protein concentration of *Mic60* and *OPA1*. Data show means ± SD (*n* = 3). * *p* < 0.05.

ELISA method was used to locate and quantify *Mic60* and *OPA1* proteins. The results from the immunohistochemical photographs showed that *Mic60* protein expressed in the myocardium of both adult and aged Tibetan sheep. *OPA1* protein expressed in the myocardium of adult Tibetan sheep, but hardly expressed in the aged sheep (Figure 2a,b,d,e). ELISA results showed that the concentrations of *Mic60* protein in the myocardium had no significant difference between adult (153.27 ± 26.28 ng/mL) and aged (150.24 ± 25.98 ng/mL) Tibetan sheep (*p* > 0.05) (Figure 2g). The concentrations of *OPA1* protein in the myocardium had significant difference between adult (49.77 ± 16.45 ng/mL) and aged (34.06 ± 12.29 ng/mL) Tibetan sheep (*p* < 0.05) (Figure 2g).

### 3.3. Mitochondrial Morphological Structure Comparison

**Figure 3:**
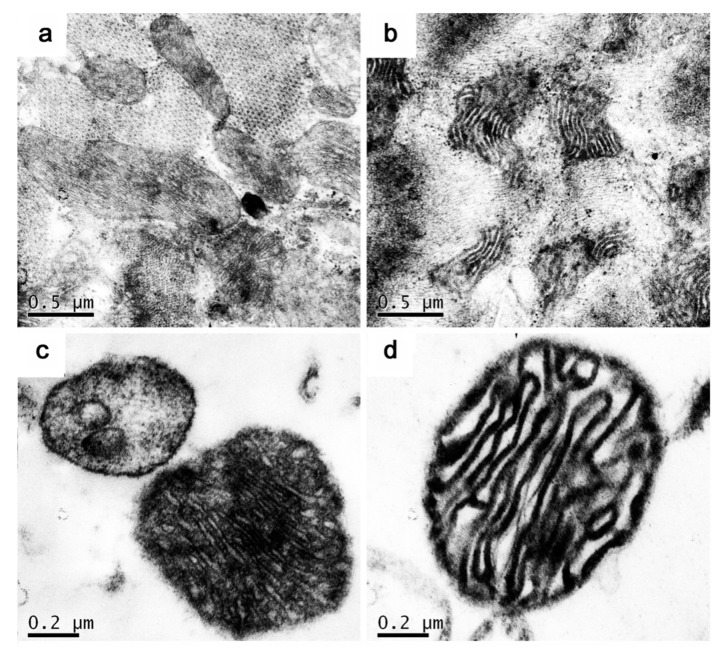
Ultrastructure observation of myocardial mitochondria in adult and aged Tibetan sheep. Note: (**a**,**b**): Adult and aged Tibetan sheep myocardial mitochondria, bar = 0.5 μm. (**c**,**d**): Mitochondria isolated from adult and aged Tibetan Sheep myocardium, bar = 0.2 μm.

**Figure 4:**
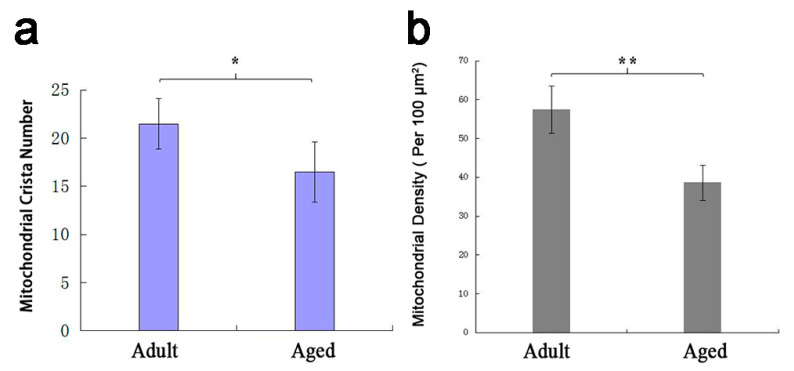
The number of mitochondrial crista (**a**) and density of mitochondria (**b**) in the myocardium of adult and aged Tibetan Sheep. Data show means ± SD. * *p* < 0.05, ** *p* < 0.01.

The mitochondrial ultrastructure of myocardium was observed by transmission electron microscopy, and the morphological and the difference of myocardial mitochondria between adult and aged Tibetan sheep were compared. The average number of mitochondrial crista and the density of mitochondria isolated were calculated. The mitochondria in the myocardium of both adult and aged Tibetan sheep had clear double membranes and tubular cristae, and their structures were complete (Figure 3a,b). The mitochondrial density in the myocardium of adult Tibetan sheep was 57.4 ± 6.07/100 μm^2^, which was significantly higher than that of the older age group (38.6 ± 4.51/100 μm^2^) (*p* < 0.01) (Figure 4b). The number of mitochondria cristae in the myocardium of adult Tibetan sheep was 21.5 ± 2.65, which was denser than that of 16.5 ± 3.11 in aged Tibetan sheep (*p* < 0.05) (Figure 4a). The mitochondria of adult were closely related but were not closely related in the aged Tibetan sheep, being slightly fragmented, and the cristae membrane were slightly swelled (Figure 3a,b).

## 4. Discussion

The expression of mitochondrial inner membrane proteins *Mic60* and *OPA1* may be related to aging. Studies found that with the *Mic60* heterozygous deletion, the expression of senescence-associated protein p21 in the myocardium of mice was significantly increased, indicated that the mitochondrial inner membrane protein *Mic60* may be related to cardiac senescence in mice [23]. Eckert et al. found that the expression of *OPA1* in mice was decreased with age [24]. A similar result was obtained by Xu et al., the expression of *OPA1* in Drosophila was significantly decreased with age [25]. In this study, the expression levels of *Mic60* and *OPA1* genes in the myocardium of aged Tibetan sheep were significantly lower than that of adult, which were 0.32 and 0.22 times of that of adult Tibetan sheep, respectively, which indicated that the expression of *Mic60* and *OPA1* genes in the myocardium of Tibetan sheep would reduce with the age. The expression of *OPA1* protein in the aged Tibetan sheep was significantly lower than that of adult. In spite of this, the expression of *Mic60* protein in the myocardium of aged Tibetan sheep was similar to that of adult, indicating that the expression of *Mic60* protein may affect by other factors, which deserved further investigation. 

In this study, the density of myocardial mitochondria in aged Tibetan sheep was significantly lower than that of adult, which indicated that myocardial mitochondrial density was age-dependent. Preston et al. reported that the adult rat heart mitochondrial density was higher than that of older rat [26]. The similar phenomenon was found in the studies of yak heart, which showed that mitochondrial density of heart gradually decreased with age, and the concentration of mitochondrial protein gradually increased [27]. Studies on the heart of yak from six months to two years old showed that the density of myocardial mitochondria gradually decreased with age [28], which was consistent with this research.

The expression of *Mic60*, *OPA1* genes and *OPA1* protein were significantly lower in aged Tibetan sheep myocardium than that of adult in this study. Besides, the number of mitochondrial crista was significantly lower, the crista membrane was wide and the mitochondria were not closely linked, showing fragmentation in aged Tibetan sheep. Von et al. reported that absence of fcj1 (Mic60 in mammals) would lead to the mitochondrial crista membrane becoming hypertrophic and stacked, the crista junction being lost and the crista separating from the inner boundary membrane in the yeast cells [16]. Studies found that the oligomerization of *OPA1* in starvation cells was significantly increased, while the crista width of the mitochondria in the starving cells were narrowed obviously and *OPA1* had a regulatory effect on mitochondrial crista independent of its fusion effect [29]. *OPA1* protein was also found to be related to the tubular network structure of mitochondria, and inactivation of *OPA1* would make the network cracked and aggravate mitochondrial fragmentation [30]. Some studies have shown that the expression of *OPA1* was positively correlated with the number of mitochondria crista. Researchers found that a large number of crista structures disappeared in mitochondria when the *OPA1* protein was inhibited in Hela cells [15]. Studies also showed that the interaction between *OPA1* and *Mic60* regulated the morphology of mitochondrial [31]. Therefore, the mitochondrial morphology of aged Tibetan sheep is different from the adult Tibetan ones, which may be influenced by the regulation of *Mic60* and *OPA1* genes and proteins expression, with *OPA1* possibly playing a more important role.

Mitochondria cristae can greatly increase mitochondrial inner membrane surface area and provide more attachment sites for oxidative phosphorylation-related proteins and enzymes. The more mitochondria cristae there are, the stronger oxidative phosphorylation ability they have, and the higher metabolic activity and energy consumption. The mitochondrial of the adult Tibetan sheep has a larger crista membrane area compared with the aged sheep. This structural feature indicates that the mitochondria of adult Tibetan sheep have stronger oxidative phosphorylation ability.

## 5. Conclusions

In conclusion, our study investigated the expression of *Mic60* and *OPA1* genes and proteins and the morphology of mitochondria in the myocardium of adult and aged Tibetan sheep. Our data showed that the expression of *Mic60*, *OPA1* genes and *OPA1* protein were significantly lower in aged Tibetan sheep myocardium than that of adult in this study. Besides, the number of mitochondrial crista was significantly lower, the crista membrane was wide and the mitochondria were not closely linked, showing fragmentation in aged Tibetan sheep. These results suggest the expression of *Mic60* and *OPA1* genes and *OPA1* protein will reduce, while reducing the capacity of myocardial mitochondria in Tibetan sheep with age.

## Figures and Tables

**Table 1:** Primer information for Tibetan sheep *MIC60*, *OPA1* and β-actin genes.

Gene	GenBank Accession No.	Primer Sequence (5′–3′)	Primer Length	Product Length
*Mic60*	XM_012169573	F:TTGAGATGGTCCTTGGTT	18	136
		R:TTGTTTCTGAGGTGGTGAG	19	
*OPA1*	XM_012140446	F:ATGAAATAGAACTCCGAATG	20	112
		R:GTCAACAAGCACCATCCT	18	
β-actin	NM_001009784	F: AGCCTTCCTTCCTGGGCATGGA	22	113
		R:GGACAGCACCGTGTTGGCGTAGA	23	

## References

[1] Dimmer K.S., Scorrano L. (2006). (De) constructing mitochondria: What for?. Physiology.

[2] Frezza C., Cipolat S., Brito O.M.D., Micaroni M., Beznoussenko G.V., Rudka R.T., Bartoli D., Polishuck R.S., Danial N.N., Strooper B.D. (2006). OPA1 Controls Apoptotic Cristae Remodeling Independently from Mitochondrial Fusion. Cell.

[3] Okamoto K., Shaw J.M. (2005). Mitochondrial Morphology and Dynamics in Yeast and Multicellular Eukaryotes. Annu. Rev. Genet..

[4] Chan D.C. (2006). Mitochondrial Fusion and Fission in Mammals. Annu. Rev. Cell. Dev. Bi..

[5] Cogliati S., Frezza C., Soriano M.E., Varanita T., Quintana-Cabrera R., Corrado M., Cipolat S., Costa V., Casarin A., Gomes L.C. (2013). Mitochondrial Cristae Shape Determines Respiratory Chain Supercomplexes Assembly and Respiratory Efficiency. Cell.

[6] Gilkerson R.W., Selker J.M.L., Capaldi R.A. (2003). The cristal membrane of mitochondria is the principal site of oxidative phosphorylation. FEBS Lett..

[7] Chaban Y., Boekema E.J., Dudkina N.V. (2014). Structures of mitochondrial oxidative phosphorylation supercomplexes and mechanisms for their stabilisation. BBA-Bioenerg..

[8] Vogel F., Bornhovd C., Neupert W., Reicher A.S. (2006). Dynamic subcompartmentalization of the mitochondrial inner membrane. J. Cell Biol..

[9] Carelli V., Morgia C.L., Iommarini L., Carroccia R., Mattiazzi M., Sangiorgi S., Farne S., Maresca A., Foscarini B., Lanzi L. (2007). Mitochondrial Optic Neuropathies: How Two Genomes may Kill the Same Cell Type?. Biosci. Rep..

[10] Gieffers C., Korioth F., Heimann P., Ungermann C., Frey J. (1997). Mitofilin is a transmembrane protein of the inner mitochondrial membrane expressed as two isoforms. Exp. Cell. Res..

[11] Olichon A., Elachouri G., Baricault L., Delettre C., Belenguer P., Lenaers G. (2007). OPA1 alternate splicing uncouples an evolutionary conserved function in mitochondrial fusion from a vertebrate restricted function in apoptosis. Cell Death Differ..

[12] Youle R.J., van der Bliek A.M. (2012). Mitochondrial Fission, Fusion, and Stress. Science.

[13] Spinazzi M., Cazzola S., Bortolozzi M., Baracca A., Loro E., Casarin A., Solaini G., Sgarbi G., Casalena G., Cenacchi G. (2008). A novel deletion in the gtpase domain of opa1 causes defects in mitochondrial morphology and distribution, but not in function. Hum. Mol. Genet..

[14] Griparic L., Kanazawa T., Van Der Bliek A.M. (2007). Regulation of the mitochondrial dynamin-like protein Opa1 by proteolytic cleavage. J. Cell Biol..

[15] Griparic L., Van Der Wel N.N., Orozco I.J., Peters P.J., Van Der Bliek A.M. (2004). Loss of the intermembrane space protein Mgm1/OPA1 induces swelling and localized constrictions along the lengths of mitochondria. J. Biol. Chem..

[16] Von der Malsburg K., Mueller J.M., Bohnert M., Oeljeklaus S., Kwiatkowska P., Becker T., Loniewska-Lwowska A., Wiese S., Rao S., Milenkovic D. (2011). Dual Role of Mitofilin in Mitochondrial Membrane Organization and Protein Biogenesis. Dev. Cell.

[17] Head B.P., Zulaika M., Ryazantsev S., van der Bliek A.M. (2011). A novel mitochondrial outer membrane protein, MOMA-1, that affects cristae morphology in Caenorhabditis elegans. Mol. Biol. Cell.

[18] Yang R.F., Sun L.H., Zhang R., Zhang Y., Luo Y.X., Zheng W., Zhang Z.Q., Chen H.Z., Liu D.P. (2015). Suppression of Mic60 compromises mitochondrial transcription and oxidative phosphorylation. Sci. Rep..

[19] Koerner C., Barrera M., Dukanovic J., Eydt K., Harner M., Rabl R., Vogel F., Rapaport D., Neupert W., Reichert A.S. (2012). The C-terminal domain of Fcj1 is required for formation of crista junctions and interacts with the TOB/SAM complex in mitochondria. Mol. Biol. Cell.

[20] John G.B., Shang Y., Li L., Renken C., Mannella C.A., Selker J.M.L., Rangell L., Bennett M.J., Zha J. (2005). The mitochondrial inner membrane protein mitofilin controls cristae morphology. Mol. Biol. Cell.

[21] Chantler P.D., Goldspink D.F., Clements R.E., Sharp L., Schlosshan D., Tan L.B. (2006). Congestive heart failure: Extent of cardiac functional changes caused by aging and organ dysfunction. Heart.

[22] Livak K., Schmittgen T. (2000). Analysis of Relative Gene Expression Data Using Real-Time Quantitative PCR and the 2-△△Ct Method. Methods.

[23] Wang C.L., Sun L.H., Yue Y.S. (2017). Correlation between Mic60 haploid insufficiency and cardiac aging in mouse. Chin. J. Pathol..

[24] Eckert G.P., Schiborr C., Hagl S., Abdel-Kader R., Muller W.E., Rimbach G., Frank J. (2013). Curcumin prevents mitochondrial dysfunction in the brain of the senescence-accelerated mouse-prone 8. Neurochem. Int..

[25] Xu C., Wang H., Wang Z., Wang Q., Zhang S., Deng Y., Fang Y. (2017). In vivo imaging reveals mitophagy independence in the maintenance of axonal mitochondria during normal aging. Aging Cell.

[26] Preston C.C., Oberlin A.S., Holmuhamedov E.L., Gupta A., Sagar S., Syed R.H.K., Siddiqui S.A., Raghavakaimal S., Terzic A., Jahangir A. (2008). Aging-induced alterations in gene transcripts and functional activity of mitochondrial oxidative phosphorylation complexes in the heart. Mech. Ageing Dev..

[27] Simonson T.S., Yang Y., Huff C.D., Yun H., Qin G., Witherspoon D.J., Bai Z., Lorenzo F.R., Xing J., Jorde L.B. (2010). Genetic evidence for high-altitude adaptation in Tibet. Science.

[28] He Y., Yu S., Hu J., Cui Y., Liu P. (2016). Changes in the Anatomic and Microscopic Structure and the Expression of HIF-1α and VEGF of the Yak Heart with Aging and Hypoxia. PLoS ONE.

[29] Patten D.A., Wong J., Khacho M., Soubannier V., Mailloux R.J., Pilon-Larose K., MacLaurin J.G., Park D.S., McBride H.M., Trinkle-Mulcahy L. (2014). OPA1-dependent cristae modulation is essential for cellular adaptation to metabolic demand. Embo J..

[30] Meeusen S., DeVay R., Block J., Cassidy-Stone A., Wayson S., McCaffery J.M., Nunnari J. (2006). Mitochondrial inner-membrane fusion and crista maintenance requires the dynamin-related GTPase Mgm1. Cell.

[31] Darshi M., Mendiola V.L., Mackey M.R., Murphy A.N., Koller A., Perkins G.A., Ellisman M.H., Taylor S.S. (2011). ChChd3, an Inner Mitochondrial Membrane Protein, Is Essential for Maintaining Crista Integrity and Mitochondrial Function. J. Biol. Chem..

